# The HHS Strategic Framework on multiple chronic conditions: genesis and focus on research

**DOI:** 10.15256/joc.2013.3.20

**Published:** 2013-12-24

**Authors:** Anand K. Parekh, Richard A. Goodman

**Affiliations:** ^1^HHS Office of the Assistant Secretary for Health; ^2^National Center for Chronic Disease Prevention and Health Promotion, Centers for Disease Control and Prevention, Atlanta, GA, USA

**Keywords:** Comorbidities, multimorbidity, multiple chronic conditions, strategic framework, research

## Abstract

Among the 21st century’s major emerging health issues, one of the most critical is the increasing prevalence of individuals with comorbidities, or multiple chronic conditions (MCCs), and the myriad challenges this poses for public health, healthcare, social services, and other sectors. Given the increasing prevalence of individuals with MCCs and the paramount role of MCCs as a healthcare cost driver, in 2008 the U.S. Department of Health and Human Services (HHS) launched an initiative to strengthen efforts by the HHS to address the effects of MCCs on health status, quality of life, and cost. In this paper, we first provide an overview of the HHS initiative with a particular focus on the approach used in developing the initiative’s centerpiece, the *HHS Strategic Framework on Multiple Chronic Conditions*; we next describe progress in implementing one of the framework’s four major goal areas (Goal 4) on facilitating research to fill knowledge gaps about, and interventions and systems to benefit, individuals with MCCs; and we conclude by suggesting additional potential priorities for research on MCCs. Although considerable research on MCCs has been reported over the past decade, the HHS Strategic Framework’s goal on research provides a set of priority areas and a plan for systematically strengthening the evidence and information foundation necessary to address the challenges of MCCs in the USA. More broadly, the Strategic Framework provides a roadmap to help improve coordination between HHS operating divisions and enhance collaboration with external stakeholders to improve the quality of life for those with MCCs.

Journal of Comorbidity 2013;3:22–29

## Introduction

Among the 21st century’s major emerging health issues, one of the most critical is the increasing prevalence of individuals with comorbidities, or multiple chronic conditions (MCCs), and the myriad challenges this poses for public health, healthcare, social services, and other sectors [1, 2]. The construct of comorbidity dates back to at least 1970, when Feinstein used the term in addressing the functional effects of comorbid conditions on the patient and diagnostic implications [3, 4]. Since that time, discussion of this issue in the literature has expanded substantially [5], and has been reflected by several other terms, including multimorbidity [6], co-occurring chronic diseases [7], multiple chronic conditions [1, 8], and other related terms, such as polypathology and pluripathology [9], and medically complex patients [10]. While the issues described by Feinstein over 4 decades ago remain in force today, comorbidity is now the principal driver for healthcare expenditures in the USA [11].

Given the substantial and increasing prevalence of individuals with comorbidities (subsequently referred to as MCCs) [12, 13], and the paramount role of MCCs as a healthcare cost driver, in 2008 the U.S. Department of Health and Human Services (HHS) launched an initiative to strengthen efforts by the HHS to address the effects of MCCs on health status, quality of life, and cost [14]. In this paper, we first provide an overview of the HHS initiative with a particular focus on the approach used in developing the initiative’s centerpiece, the *HHS Strategic Framework on Multiple Chronic Conditions*, and its vision of optimum health and quality of life for individuals with MCCs [8]. We next describe progress in implementing one of the framework’s four major goal areas (Goal 4) on facilitating research to fill knowledge gaps about, and interventions and systems to benefit, individuals with MCCs. We conclude by suggesting additional potential priorities for research on MCCs.

## The HHS initiative on MCCs and development of the *HHS Strategic Framework on MCCs*

The HHS initiative on MCCs dates to the fall of 2008, when the HHS Assistant Secretary for Health convened and charged a departmental workgroup to identify HHS options for improving the health of the MCC population. Since the workgroup’s creation, nearly all HHS operating divisions have been participating. The workgroup’s initial major effort was to identify ongoing HHS programs, activities, and initiatives focused on improving the health of individuals with MCCs. The resulting inventory, first released in March 2009, contained more than 50 efforts across the HHS directed primarily to the healthcare needs of people with two or more chronic health conditions. In addition, a series of interagency workgroup meetings addressed focal topics and issues, such as reducing rehospitalizations and adverse drug events in this population. The workgroup also assisted the HHS in both health reform and comparative effectiveness research efforts related to MCCs.

It soon became clear to the workgroup that, among other beneficial effects, an HHS departmental level strategic framework for improving the health status of individuals with MCCs could serve as a comprehensive roadmap for strengthening coordination within the HHS and with external stakeholders in implementing the considerable work already directed toward MCCs. The workgroup considered a variety of approaches to the complex set of issues involving MCCs and transforming those issues into a manageable plan. The result was the workgroup’s creation of a draft strategic framework consisting of four interdependent major goal areas: Goal 1, strengthening the healthcare and public health systems; Goal 2, empowering the individual to use self-care management; Goal 3, equipping care providers with tools, information, and other interventions; and Goal 4, supporting targeted research about individuals with MCCs and effective interventions [15]. Each of the goals, in turn, enumerated a set of objectives and strategies to further guide specific actions.

The draft framework underwent iterative review within the HHS’s major operating divisions, and then, because the HHS recognized that stakeholder and community involvement would be essential to the framework’s successful implementation, in May 2010, an HHS notice in the *Federal Register* invited interested parties to review and comment on the draft strategic framework and to provide feedback [16]. As a result, the HHS received, and the workgroup reviewed, over 250 comments from the public and stakeholder organizations that were carefully considered and used to improve the final product. These comments were particularly helpful in ensuring inclusion of behavioral concerns (e.g. mental illnesses, substance use, and addiction disorders) among the spectrum of health conditions the framework addressed.

In December 2010, the HHS released the *HHS Strategic Framework on Multiple Chronic Conditions* in an open conference call with over 400 registered participants. Notably, the National Council on Aging, a private sector partner, participated in the event and committed to lead an effort to help implement the Strategic Framework’s second goal on maximizing the use of self-care management. The occasion of the HHS’s release of the Strategic Framework also was announced through a press release that garnered wider attention [17–19].

Since the Strategic Framework’s release in late 2010, the HHS has maintained a sustained focus on implementing key elements in each of the four main goals [20]. For example, buoyed by the Patient Protection and Affordable Care Act, two HHS operating divisions – the Centers for Medicare and Medicaid Services (CMS) and the Substance Abuse and Mental Health Services – have helped to implement Goal 1 by testing new models of care coordination and integration for individuals with MCCs [21, 22]. In support of Goal 2, the Administration on Community Living (ACL) has taken steps to help bring the Chronic Disease Self-Management Program to scale across the USA; to date, ACL-funded states and their partners have reached over 164,000 individuals in whom MCCs are prevalent with this evidence-based program [23]. In addition, as noted above, the National Council on Aging officially launched the Self-Management Alliance, a consortium of health plans, business, and pharmaceutical companies, foundations, and federal partners which established the goal of integrating self-management within the healthcare system by 2020 [24].

Helping to advance Goal 3 and with HHS support, the National Quality Forum created a measurement framework to spur the development of quality measures applicable to the MCC population [25], and the Institute of Medicine, in conjunction with the HHS, convened clinical practice guideline developers and other experts to identify options for enhanced incorporation of common comorbidities in clinical guidelines [26]. As an additional example of Goal 3 implementation, in March 2013, the HHS released an on-line expanded inventory of the HHS and private sector-led programs, activities, and initiatives directed at improving the health of individuals with MCCs. The inventory provides basic information about 250 such activities that supported the Strategic Framework’s goals, objectives, and strategies [27]. Of these, 15 were highlighted in a separate Innovative Profiles report that emphasized features such as innovative approaches, demonstrable impact, potential for scalability, or valuable insights and lessons for professionals or individuals living with MCCs or their caregivers [28]. Finally, several efforts have been undertaken to implement Goal 4 objectives on enhancing research to fill knowledge gaps about individuals with MCCs as detailed below.

## Progress in implementing Goal 4 on research to benefit individuals with MCCs

The Strategic Framework’s Goal 4 focuses on the imperative for research to fill knowledge gaps about, and on interventions and systems to benefit, individuals with MCCs. This cross-cutting goal reinforces other parts of the Strategic Framework that call for improved information to better understand MCCs, and for expanding the evidence base for preventing and mitigating the burden of MCCs. This goal’s overall aim is expressed through four objectives that encompass, respectively, four domains: (A) increasing the external validity of clinical trials; (B) increasing understanding of the basic epidemiology of MCCs; (C) increasing research on community and patient-centered health outcomes; and (D) improving understanding of the roles of disparities in MCC populations [8].

Objective A and its strategies focus on the need for increasing the external validity of relevant clinical trials, especially by assessing the inclusion of individuals with MCCs and by reducing the unnecessary exclusion of such individuals from clinical trials. Investigators external to the HHS who cited to the framework examined whether patients with MCCs are under-represented in randomized controlled trials that are published in high-impact journals; they observed that, among the sample of reports they studied, few trials in the past 15 years included patients with MCCs [29]. Other investigators who assessed the inclusion of people with comorbidities in trials similarly note the limited attention accorded to comorbidities in clinical trials [30]. Implications surrounding this issue were shared with a broad segment of healthcare consumers by the AARP through its analysis that cited the HHS Strategic Framework and included a set of potential options [see reference 31 for these options].

Within the HHS, a study sponsored by the Food and Drug Administration (FDA) and the Assistant Secretary for Planning and Evaluation used New Drug Applications and Biologic License Applications submitted during fiscal year 2010 to examine the question of whether individuals with MCCs are being excluded from controlled clinical trials [32]. The investigators reported that, in this database population, the prevalence of several chronic conditions was lower overall than that reported in the total population, and that the two conditions most commonly excluded were hepatic disorder and psychiatric disorder. It also was stated that an analysis of the study level inclusion–exclusion criteria showed substantial variation in the number of exclusions per study by therapeutic indication. Several studies of individual chronic conditions had, on average, ten or more exclusions. At the same time, however, the investigators described a litany of methodologic challenges within the practical constraints of this study, including, for example, that the clinical trials database precluded assessing the profile of potential subjects who are screened, but excluded due to MCCs. One implication of this study is that subsequent efforts might consider additional or alternative research strategies in determining whether individuals with certain comorbidities are being unnecessarily excluded from trials. The FDA is currently reviewing the results of the study to guide its future policies and procedures in this area.

Objective B and its strategies concentrate on understanding the epidemiology of MCCs. In recent years, the number of epidemiologic studies that have examined MCC patterns among national-level population samples in the USA has been growing, as the importance of MCC populations has been realized. Examples include the report by Wolff and colleagues on MCCs in a 1999 sample of claims data for Medicare beneficiaries [1], a study of mortality rates across patients with MCCs among a cohort of US veterans who used the Veterans Health Administration services in 1999 and 2001 [33], the Robert Wood Johnson Foundation chart book on chronic care, which analyzed data from the Household Component of the 2006 Medical Expenditure Panel Survey [34], the report from the “Faces of Medicaid Data Series” that used claims data from 2001 and 2002 to identify subgroups of Medicaid beneficiaries for targeted clinical services [35], and a study of multimorbidity patterns in elderly veterans during 2007–2008 [36]. Other descriptions of MCCs among individuals in the USA have been produced by studies using data sources in individual states, such as those by Koroukian et al. [37] and by Miller et al. [38].

To help advance Objective B strategies on basic epidemiologic research and on determining MCC patterns in Medicare and other populations, the HHS formed an MCC data workgroup with experts from three agencies (the Agency for Healthcare Research and Quality [AHRQ], Centers for Disease Control [CDC], and CMS). This workgroup has conducted coordinated, parallel epidemiologic analyses of five national-level data sets encompassing populations in a spectrum of settings, including the National Health Interview Survey (NHIS) [12]; National Ambulatory Medical Care Survey [39]; Medical Expenditure Panel Survey [40]; Nationwide Inpatient Sample of the Healthcare Cost and Utilization Project [41]; and Medicare beneficiary enrollment and claims administrative data [42]. Reports on these analyses have been published together in *Preventing Chronic Disease* [43], along with reports on a conceptual model for standardizing approaches to defining, identifying, and using information about chronic conditions in the USA [44], and on a study of NHIS data to characterize co-occurring leading lifestyle-related chronic conditions among adults [13].

In addition to the coordinated analyses of five HHS data systems, since the Strategic Framework’s release in 2010, several other HHS research studies, reports, and information resources on MCCs have been made public. These include an analysis of NHIS data to compare estimates of MCC occurrence among adults aged 45 years or older during two periods of time, 1999–2000 and 2009–2010 [45]; and the CMS 2011 and 2012 chart books on chronic conditions among Medicare fee-for-service beneficiaries with a dominant emphasis on MCC prevalence, associated healthcare utilization, and costs in 2008 and 2010, respectively [11, 46]. The CMS also recently released two public-use data resources for researchers and others, including data on state level MCC prevalence, healthcare utilization, and costs among Medicare beneficiaries [47], and an interactive data dashboard allowing for examination of state- and hospital referral region (HRR)-level MCC patterns among Medicare beneficiaries [48–51]. The release of the public-use data resource on state-level MCC patterns was also followed by preparation of a report for publication [52]. In addition, the AHRQ reported findings of a study that focused on healthcare access and expenditures among non-elderly adults with MCCs by insurance coverage status during 2007–2008 [53].

Objective C and its strategies target increased clinical, community, and patient-centered research. MCC-related research initiatives supported by the National Institutes of Health (NIH) have supported several strategies under this objective. For example, during 2010–2013, the NIH announced these and other funding opportunities to foster extramural research about MCCs:

- The first, Notice number NOT-OD-11-020 (December 2010), addressed the need for more effective behavioral treatments for patients with MCCs, and encouraged investigators to address more effective research methods and measures for conceptualizing, triaging, and assessing the health behavior (e.g. adherence, mental health problems, diet and exercise, and substance use/abuse disorders) of patients with MCCs [54].
- The second, Funding Opportunity Announcement (FOA) Number PA-12-024 (November 2011), sought proposals to use a common conceptual model to develop behavioral interventions – using a common approach rather than behavior-specific interventions and/or condition-specific interventions – to improve health outcomes in patients with MCCs. Specifically, this announcement offered support for research in primary care that uses a multidisease-care management approach to behavioral interventions with high potential impact to improve patient-level health outcomes for individuals with three or more chronic health conditions [55].
- The third, FOA Number RFA-AG-13-003 (July 2012), supported secondary analyses of data sets aimed at: assessing the public health and health-cost impact of specific combinations of MCCs in defined older populations; identifying differences in effectiveness and safety of different treatment regimens for patients with specific combinations of MCCs; examining alterations in safety or effectiveness of a treatment for one condition related to the presence of one or more specific coexisting conditions; and addressing methodologic issues relevant to analyses of the health impact or treatment of MCCs [56].
- The fourth, FOA PA-13-168 (March 2013), also supported secondary analyses of data sets, with research objectives including effects of specific combinations of two or more comorbid conditions or combinations of medications on risks for specific beneficial and/or adverse health outcomes; public health and cost impact of specific combinations of two or more chronic conditions; differences in health outcomes between alternative treatment regimens or healthcare management strategies for older patients with specific combinations of two or more chronic conditions; and interactions among medications, disease processes, and health outcomes in complex older patients with MCCs [57].
- The fifth, RFA RM-13-012 (August 2013), sought applications for demonstration projects across two or more healthcare systems for efficient, large-scale pragmatic clinical trials focused on management of patients with MCCs [58].

Several other HHS developments are helping to further the Objective C focus on clinical, community, and patient-centered research that elucidates the evidence base for preventing and treating individuals with MCCs. For example, the AHRQ created the “MCC Research Network” to foster improved understanding about interventions that provide the greatest benefit to MCC patients [59]. The network includes 45 grantees with funding to advance the field of MCC research with focuses on comparative effectiveness, quality improvement, and patient-centered outcomes research. In addition, a meeting convened jointly in early 2013 by AHRQ, NIH, and the Patient-Centered Outcomes Research Institute (PCORI) examined the context of health in individuals with MCCs. The meeting’s key long-term goal – to enhance the inclusion of contextual factors in research on MCCs – was addressed by exploring key contextual factors and relevant research methods, and developing a research agenda [60; Stange KC: personal communication].

Objective D and its strategies address disparities in MCC populations, including stimulating and using research about the roles of disparities in MCC populations that would assist in focusing intervention. The HHS’s principal research effort on disparities in MCC populations is a study supported by the Office of the Assistant Secretary for Planning and Evaluation that aims to describe data systems and data sets that can be analyzed to better improve understanding of and approaches to addressing disparities in MCC populations. This effort especially focuses on describing MCC combinations that are most important for targeting interventions.

## Conclusion

Although considerable research on MCCs has been reported over the past decade, the HHS Strategic Framework provides a set of priority areas and a plan for systematically strengthening the evidence and information foundation necessary to address the challenges of MCCs in the USA. In the relatively short period since the Strategic Framework was released, HHS agencies and programs have used the framework to guide new research initiatives on MCCs. As a result, and as reflected in this report, HHS programs have pursued a broad spectrum of MCC research activities addressing priority areas by, for example, examining the inclusion (or exclusion) of comorbidities in clinical trials; conducting epidemiologic research and reporting findings on MCC occurrence in representative national-level population samples in diverse settings; supporting studies to improve the quality and effectiveness of medical management of individuals with MCCs; and increasing understanding of the effects of disparities in MCC populations.

In addition to research efforts that are already completed or still in progress, the HHS Initiative on MCCs and its derivative Strategic Framework have helped to highlight other priority focus areas for research. Among these priorities are needs for developing the following:

- Better data on, identification, and characterization of combinations of comorbidities that are prevalent, account disproportionately for suffering and healthcare expenditures, and are most amenable to interventions – this priority would extend work already in progress by refining identification of the most important dyads, triads, and other combinations of comorbidities from the standpoint of amenability to prevention, management, and mitigation.
- Expanded local-level (i.e. subnational level) data on MCCs – building on the concept of the CMS’s publicly available dashboard with state- and HRR-level data – for use in health-policy making, planning, service delivery, and program evaluation.
- More data tailored to special uses, such as helping to better inform the development of clinical practice guidelines by addressing comorbidities to guidelines’ index conditions [61–64].

Substantial efforts are being made nationally to improve the health and quality of life for individuals with MCCs. Significant gaps exist in the optimal approach to care, and these are beginning to be addressed through the research directions articulated in the Strategic Framework. More broadly, the Strategic Framework provides a roadmap to help improve coordination between HHS operating divisions and enhance collaboration with external stakeholders to improve the quality of life for those with MCCs. Now is the time to view person-centered chronic disease prevention and care management through the prism of MCCs.
